# Which Are the Best Regimens of Broad-Spectrum Beta-Lactam Antibiotics in Burn Patients? A Systematic Review of Evidence from Pharmacology Studies

**DOI:** 10.3390/antibiotics12121737

**Published:** 2023-12-14

**Authors:** Gianpiero Tebano, Giulia la Martire, Luigi Raumer, Monica Cricca, Davide Melandri, Federico Pea, Francesco Cristini

**Affiliations:** 1 Infectious Diseases Unit, Ravenna Hospital, AUSL Romagna, 48100 Ravenna, Italy; 2 Infectious Diseases Unit, Forlì and Cesena Hospitals, AUSL Romagna, 47121 Forlì and Cesena, Italy; 3 Unit of Microbiology, The Greater Romagna Area Hub Laboratory, 47522 Cesena, Italy; monica.cricca3@unibo.it; 4 Department of Medical and Surgical Sciences (DIMEC), Alma Mater Studiorum, University of Bologna, 40126 Bologna, Italy; davide.melandri@auslromagna.it (D.M.); federico.pea@unibo.it (F.P.); 5 Dermatology Unit and Burn Center, AUSL Romagna, Cesena Hospital, 47521 Cesena, Italy; 6 Clinical Pharmacology Unit, IRCCS Azienda Ospedaliero Universitaria di Bologna, 40138 Bologna, Italy

**Keywords:** burns, clinical pharmacology, pharmacokinetics, pharmacodynamics, anti-bacterial agents

## Abstract

Background: Burn injury causes profound pathophysiological changes in the pharmacokinetic/pharmacodynamic (PK/PD) properties of antibiotics. Infections are among the principal complications after burn injuries, and broad-spectrum beta-lactams are the cornerstone of treatment. The aim of this study was to review the evidence for the best regimens of these antibiotics in the burn patient population. Methods: We performed a systematic review of evidence available on MEDLINE (from its inception to 2023) of pharmacology studies that focused on the use of 13 broad-spectrum beta-lactams in burn patients. We extracted and synthetized data on drug regimens and their ability to attain adequate PK/PD targets. Results: We selected 35 studies for analysis. Overall, studies showed that both high doses and the continuous infusion (CI) of broad-spectrum beta-lactams were needed to achieve internationally-recognized PK/PD targets, ideally with therapeutic drug monitoring guidance. The most extensive evidence concerned meropenem, but similar conclusions could be drawn about piperacillin-tazobactam, ceftazidime, cefepime, imipenem-clinastatin and aztreonam. Insufficient data were available about new beta-lactam-beta-lactamase inhibitor combinations, ceftaroline, ceftobiprole and cefiderocol. Conclusions: Both high doses and CI of broad-spectrum beta-lactams are needed when treating burn patients due to the peculiar changes in the PK/PD of antibiotics in this population. Further studies are needed, particularly about newer antibiotics.

## 1. Introduction

Burn patients are a peculiar population of critically ill patients. Burn injury, particularly when involving more than 20% of total body surface area (TBSA), produces extensive and dynamic pathophysiological changes. These include: dramatic variation of hemodynamic status, altered fluid balance, altered protein homeostasis, evolving changes of volume of distribution (Vd), and increased total and renal clearance (CL_T_ and CL_R_); all of these exhibit substantial inter-patient variability [1,2,3,4,5]. Moreover, such burn patients require long hospital stays and need intensive health care support, with a consequent high incidence of hospital-acquired infectious complications. In patients who survive the immediate post-burn resuscitation phase, infections are the main cause of death [6,7,8].

Multi-drug resistant (MDR) gram-negative bacteria (particularly *Pseudomonas aeruginosa*, *Acinetobacter baumannii* and *Enterobacterales*) are among the most frequently involved pathogens in post-burn infections. Consequently, broad-spectrum beta-lactam antibiotics are the cornerstone of antibiotic treatment in this patient population [9,10]. Because of the aforementioned pathophysiological alterations following severe burn injuries, many pharmacokinetic (PK) parameters of this class of antibiotics can differ in burn patients compared with the general patient population [2]. Specifically, it has been shown that beta-lactam antibiotics have an increased Vd and CL_T_ in burn patients [11,12,13]. For these reasons, conventional antibiotic dosages and administration modes may be inadequate to achieve optimal pharmacokinetic/pharmacodynamic (PK/PD) targets.

It has been suggested that therapeutic drug monitoring (TDM) should guide antibiotic use in the post-burn setting, particularly in more severely ill patients or in those who have augmented renal clearance (ARC) or who are affected by pathogens with borderline susceptibility in terms of minimum inhibitory concentrations (MICs) [2,14,15,16,17]. However, the interpretation of existing evidence about clinically efficacious antibiotic regimens is particularly challenging. In fact, current evidence on optimal PK/PD targets is not sufficient to recommend a single target for each antibiotic [18,19,20]. Recently Abdul-Aziz et al. [21] published a position paper proposing a set of PK/PD indexes that included data from pre-clinical and clinical studies; they reported both targets for efficacy and thresholds for toxicity. This work was carried out by some of the leading experts in the field and was endorsed by several eminent scientific societies. Very recently Hong et al. [20] published a set of recommendations for the use of beta-lactam antibiotics via prolonged infusion in critically ill patients; they suggested more aggressive PK/PD targets for beta-lactams to prevent the risk of development and selection of antibiotic resistance. The variability of suggested PK/PD targets in the literature makes it difficult to generalize the findings of different studies.

The aim of this review was to perform a systematic analysis of the available evidence concerning the PK and PD aspects of broad-spectrum beta-lactam antibiotics in burn patients, with a particular focus on drug regimens and the probability of attaining optimal PK/PD targets.

## 2. Results

**Figure 1:**
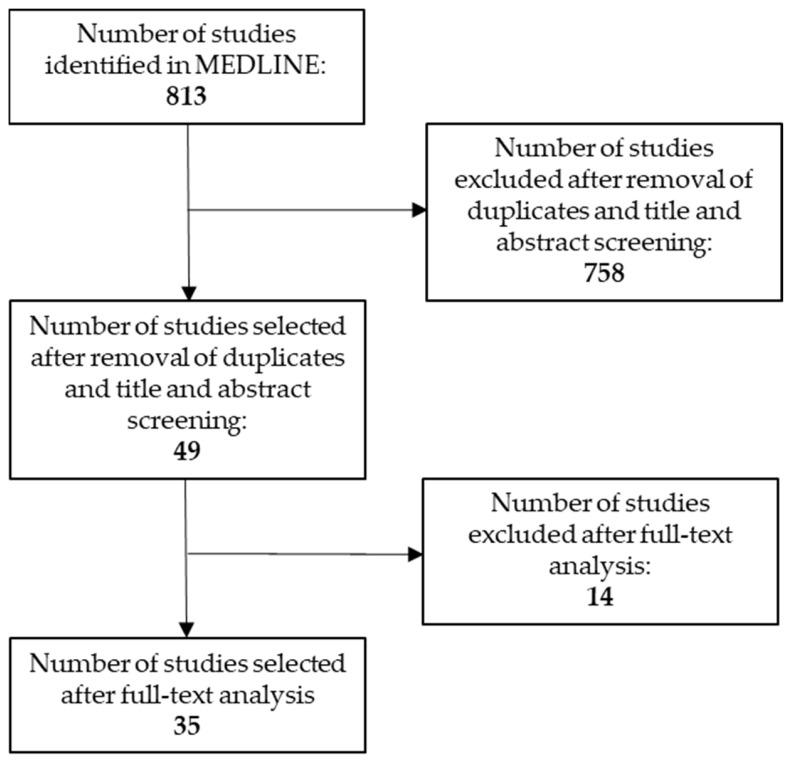
Flow chart of the study.

The initial literature search identified 813 relevant papers. After title and abstract (TIAB) screening, we selected 49 papers. Finally, after full text analysis, 35 papers were selected for final analysis (Figure 1, Table 1, Appendix A). The results for each antibiotic are synthetized in Table 1 and Table 2 and detailed in the following paragraphs.

### 2.1. Ceftazidime

The initial literature search found 168 studies, and 7 were selected after TIAB screening and full-text analysis. Dailly et al. [32] and Conil et al. [27] described the PK of ceftazidime with a population pharmacokinetic approach; however, they did not suggest any specific drug regimen in these two papers. Conil et al. addressed this issue in two other studies [26,28]. In the first, [26] they studied the PK of ceftazidime in a group of 17 burn patients who received 1 g every 4 h via intermittent infusion (IIn). As expected, they showed that the main PK parameter affected by burns was CL_R_; moreover, they found that, given an MIC of 4 mg/L, the Cmin (trough concentration)/MIC ratio was lower than 4 in 58% of patients. In order to achieve this aggressive PK/PD target, they therefore suggested either intensifying the dosing frequency or using continuous infusion (CI). The same group of authors confirmed these findings in another study [28]. In this work, they randomly assigned burn patients to receive ceftazidime either 1 g every 4 h or 2 g every 8 h via Iin; they considered Cmin > 4 × MIC the desired PK/PD target. They showed a low probability of target attainment (PTA) (50% and 20% for the 2 regimens, respectively), suggesting that either intensified administration or CI should be the preferred treatment choices. In a more recent paper, the same group [26] evaluated a population PK model testing several discontinuous and continuous regimens of ceftazidime. They concluded that a regimen ranging from 3 to 16 g/day was needed (depending on CL_R_ and age), preferably via CI, in order to achieve a target of 100% fT > MIC (duration of time that the free drug concentration remains above the MIC during a dosing interval) or greater against susceptible pathogens with an MIC up to a value of 8 mg/L. A study by Le Floch et al. [38] confirmed that CI attained a fT > 4 × MIC target (duration of time that the drug concentration remains 4 times above the MIC during a dosing interval) in 77% of patients, although no details about MICs and dosing regimens were reported. Finally, Walstad et al. [49] showed good tissue and burn blister fluid concentrations of ceftazidime after two doses of 1 g every 8 h via IIn.

#### Synthesis of the Findings concerning Drug Regimens for Ceftazidime

Evidence for the use of ceftazidime in burn patients is quite limited and came mostly from one study group. After a loading dose, 1 g every 4 h via Iin, or preferably 6 g/24 h via CI, should be sufficient to achieve a target of 60–100% fT > MIC [21], particularly in the presence of pathogens with borderline susceptibility.

### 2.2. Cefepime

The initial literature search found 51 studies, and 5 were selected after TIAB screening and full-text analysis. In a previously mentioned study, Conil et al. [26] also studied the PK of cefepime in a group of 13 burn patients who received 2 g every 8 h via IIn. As with ceftazidime, they showed that the main PK parameter of cefepime in patients affected by burns was CL_R_. Given an MIC of 4 mg/L, they found a Cmin/MIC ratio lower than 4 in 80% of patients. Thus, they suggested either further shortening the dosing interval or using CI. With a less strict target from a single case report, Aoki et al. [23] suggested shortening the dosing interval as well. In contrast, in a less recent study on 6 burn patients, Sampol et al. [44] argued that a regimen of 2 g every 12 h should be sufficient, as they did not find any relevant differences in Cmax, t1/2, Vd or CL_T_ in their burn patients compared with historical data on healthy volunteers. However, they did not consider any specific PK/PD target, and the mean trough concentration they found was lower than 2.5 mg/L at 12 h, making it unlikely that this regimen would reach an aggressive PK/PD target (such as 100% fT > MIC) in the presence of a pathogen showing borderline susceptibility. Bonapace et al. [11] performed a PK analysis on 12 burn patients after a single 2 g dose of cefepime. In this sample, where patients had a mean % of burned TBSA of 35%, they found that both mean CL_CR_ (135 mL/m) and Vd were increased. However, when normalized by CL_CR_, CL_T_ and CL_R_ were similar to those previously reported for healthy volunteers. Moreover, they estimated that different regimens (1 g every 8 h, 2 g every 12 h or 2 g every 8 h, administered over 30 min) were sufficient to achieve a target of 60% T > MIC with MIC values ≤ 8 mL/L. In addition, Alshaer et al. [22] recently performed a before/after study investigating the implementation of TDM in a burn unit. They administered 6 g of cefepime (mean values not specified at patient level) in 42 infectious episodes and found an overall PTA ranging from 60% to 100%, according to different targets. However, they referenced a Cmin > MIC target, which is a less consolidated PK/PD index for cefepime compared to fT > MIC.

#### Synthesis of the Findings concerning Drug Regimens for Cefepime

Evidence for the use of cefepime in burn patients is very limited, both in terms of the number of studies and the number of patients included. To achieve a target of 60–100% fT > MIC [21], a dose of 2 g of cefepime every 8 h should be sufficient. However, after a loading dose a CI of 6 g/24 h may be more appropriate, particularly when treating severe infections or when strains have higher MICs.

### 2.3. Piperacillin-Tazobactam

The initial literature search found 127 studies, and 10 were selected after TIAB screening and full-text analysis. In 1990, Shikuma et al. [47] initially described the principal PK parameters of piperacillin-tazobactam in 9 severely burned patients, showing a remarkable inter-patient variability and demonstrating the impact of patients’ pathophysiologic and metabolic changes after burn injury. Bourget et al. [25] reported that a dose of 4.5 g every 6 h via IIn was able to achieve a target of 100% T > MIC in a sample of 10 burn patients affected by *P. aeruginosa*, *Enterobacterales* and streptococcal infections. In this study, which included patients with a mean of 41% burned TBSA and a mean CL_CR_ of 120 mL/m, this regimen resulted in Cmins at day 1 and day 3 higher than the breakpoints of the involved bacteria, including the 16 mg/L of *P. aeruginosa*. More recently, Jeon et al. [37] reported some less encouraging data obtained from a population PK model based on 5 burn patients. The results are difficult to compare with those of the previous study [25] because of the different methodology and regimen (4.5 g every 8 h over 30 min). However, it is worth noting that they suggested a more lenient PK/PD target (50% fT > MIC); additionally, they found that the PTA against the MIC distributions of the strains reported in the EUCAST database was only 85.2% for *E. coli* and 72.3% for *K. pneumoniae*. Thus, they suggested increasing the frequency of administration and/or the duration of the infusion, particularly for patients with ARC. Olbrisch et al. [40] performed an observational comparative study, which included 20 burn patients and 16 ICU patients, that also employed an ancillary population PK model. Given 2 PK/PD targets (100% fT > MIC and 100% fT > 4 × MIC), they demonstrated that when administering a regimen of 4.5 g every 8 h over 30 min, the PTA against pathogens with an MIC of 16 mg/L was as low as 55% and 17%, respectively. These values were much lower compared to those achieved in other types of ICU patients. Based on data obtained from 2 patients and data from their population PK model, they suggested that the daily dose might need to be increased up to 27 g/24 h via prolonged infusion. The need for higher doses was also reported by Torian et al. [48] in a case report of an obese burn patient with ARC. Another comparative study recently performed by Selig et al. [45] described a population PK model evaluated in 5 burn patients and 14 patients with other types of traumas. They did not report significant discrepancies in the two samples; they concluded that, given MIC values of 8 and 16 mg/L, the standard regimen of 4.5 g every 6 or 8 h over 30 m was unable to achieve a reliable PTA, even given a low PK/PD target of 50% fT > MIC. Conversely, dosing regimens of 13.5 and 18 g/24 h via CI resulted in a 100% fT > MIC but not a 100% fT > 4 × MIC. However, it is worth highlighting that this study sample had a high mean weight (88 kg) and a very high mean CL_CR_ (177 mL/m).

Four papers [14,15,22,41] reported monocentric experiences of the implementation of TDM in guiding antibiotic treatment in burn patients, including piperacillin or piperacilin-tazobactam. Patel et al. [41] reported using TDM in a sample of 50 burn patients, which included 6 patients treated with 4.5 g piperacillin-tazobactam every 6 h. Given an MIC of 2 mg/L, they reported attaining a 100% fT > 4 × MIC target in 2/6 patients (34%). Machado et al. [15] reported clinical outcomes after implementing TDM and PK parameters, but they did not provide any information detailing drug regimens or PTA in the group not treated via TDM. In a randomized controlled trial that included 38 burn patients, Fournier et al. [14] reported that 68% of patients in the group whose treatment was not guided via TDM achieved a PK/PD target of 100% fT > MIC. They used a regimen of 4.5 g every 8 h, over 30 min or 2 h, and included infections caused by different bacterial species, including *P. aeruginosa*. Finally, Alshaer et al. [22] showed that the use of TDM was associated with an increased PTA, but they did not give detailed information about the drug regimens associated with target attainment.

#### Synthesis of the Findings concerning Drug Regimens for Piperacillin-Tazobactam

Evidence for the use of for piperacillin-tazobactam in burn patients is limited. After a loading dose, a dosing regimen of 18 g/24 h (16 g of piperacillin component, 2 g of tazobactam component) via CI should be sufficient for attaining a PK/PD target of 50–100% fT > MIC [21]. Higher TDM-guided doses may be needed in patients with ARC.

### 2.4. Meropenem

The initial literature search found 131 studies, and 11 were selected after TIAB screening and full-text analysis. In a pivotal study, Doh et al. [33] developed a population PK model of meropenem based on 59 burn patients with a mean of 49% burned TBSA. They chose *P. aeruginosa* strains isolated from Korea as target pathogens, had a median MIC < 4 mg/L and assumed a lenient target of 40% fT > MIC. They showed that a standard dose of 1 g every 8 h over 30 min was insufficient; the PTA increased up to approximately 70% with a dose of 1 g every 8 h over 3 h. It is relevant to emphasize that this study sample had a moderate ARC (mean CL_CR_ 138 mL/m) and a very low mean body weight (66 kg). The need for higher doses and CI was confirmed in two more recent studies. In the first, Ramon-Lopez et al. [43] developed a population PK model that included 12 burn patients and examined different targets, ranging from 40% T > MIC to 80% T > MIC. They showed that a standard dose of 1 g every 8 h over 30 min would be sufficient only for pathogens with low MICs, whereas a 6 g/24 h dose by prolonged infusion or CI would be more appropriate for bacteria with higher MICs. Selig et al. [46] chose a lenient target of 40% fT > MIC and evaluated a population PK model that included 11 burn patients. They showed that a standard dose of 1 g every 8 h over 30 min or over 3 h was sufficient only in burn patients without ARC or those affected by pathogens having an MIC < 4 mg/L. Continuous infusion improved PTA in patients who had ARC or infections with higher MICs. Messiano et al. [39] reported 90 meropenem concentrations from 15 burn patients. They assumed a target of 100% fT > MIC and administered 1 g meropenem every 8 h over 3 h, reporting a target attainment > 80% only for MICs ≤ 2 mg/L. Comparable results were reported by Corcione et al. [16], who studied 17 burn patients, assumed a target of 75% fT > MIC and evaluated a population PK model simulating PTA at different MIC values. They found that 76% of patients achieved the target with an MIC ≤ 2 mg/L.

Meropenem was also examined in 4 studies focusing on the effect of TDM use in burn patients [14,15,22,41]. The randomized controlled trial by Fournier et al. [14] aimed at determining the impact of TDM in a sample of 38 burn patients and also included some patients treated with meropenem. The drug regimen was 1 g every 8 h over 30 min or 2 h. In the subgroup of patients whose treatment was not guided by TDM, the overall PTA for meropenem (target: 100% fT > MIC) was 56%, but it decreased to 22% among patients with > 40% of burned TBSA (this latter finding applied to all administered antibiotics and was not available for meropenem only). The PTA increased under treatment guided by TDM. Machado et al. [15] and Alshaer et al. [22] performed two before/after studies on the implementation of TDM that included patients on meropenem, but they did not provide information regarding which drug regimens were better associated with target attainment. Patel et al. [41] reported on using TDM in 50 burn patients. Among these, only 1 patient was treated with meropenem, and this patient did not achieve 100% fT > MIC after a 1 g every 8 h by IIn dosing regimen.

Finally, there were also two case reports. Hallam et al. [36] reported the case of an extensively burned patient (52%) who did not attain a 100% fT > MIC for an MIC of 4 mg/L, with a dose of 1 g every 4 h (given via IIn). Cotta et al. [30] reported the case of a patient with extensive burns, severe ARC and infection caused by *P. aeruginosa* with a high MIC (8 mg/dL), in whom the dosing regimen of 2 g every 6 h over 3 h achieved only lenient targets.

#### Synthesis of the Findings concerning Drug Regimens for Meropenem

Evidence for the use of meropenem in burn patients is more abundant compared with other beta-lactams. After a loading dose, a dosing regimen of 6 g/24 h by CI should be sufficient to achieve a target of 50–100% fT > MIC [21]. Higher TDM-guided doses may be needed for patients with ARC or MICs > 4 mg/L.

### 2.5. Imipenem-Cilastatin

The initial literature search found 270 studies, and 8 were selected after TIAB screening and full-text analysis. In 1990, Boucher et al. [24] described the PK of imipenem-cilastatin in a sample of 11 burn patients treated with a standard regimen of 500 mg every 6 h via IIn. They reported PK parameters that were similar to those previously reported in healthy volunteers; however, the sample also included patients with second degree burns. CL_CR_ was closely related to imipenem-cilastatin CL. In a study that included 47 patients, Dailly et al. [31], confirmed the relationship between CL_CR_ and the CL of imipenem-cilastatin. Gomez et al. [35] performed a clinical PK study associated with a population PK model based on 51 burn patients. Their sample included 36 patients with normal renal function and 15 patients with renal failure (CL_CR_ was not specified) with a mean burned TBSA of 36%. They assumed a lenient PK/PD target of 40% fT > MIC and showed that mean daily doses of approximately 2 g for patients with normal renal function and 1 g for patients with renal failure (further regimen details were not provided) were able to achieve nearly 100% PTA for MICs ≤ 4 mg/L. Le Floch et al. [38] reported their results using 120 concentration measurements of imipenem-cilastatin in burn patients, showing that Cmin was never higher than 6 mg/L when a mean total daily dose of 3 g was administered. However, the lack of some data made interpretation difficult. 

Three studies explored the PK of imipemen/cilastatin in burn patients undergoing continuous renal replacement therapy (CRRT). Boucher et al. [24] described the PK of imipenem-cilastatin in a sample of 10 burn patients undergoing CRRT and treated (9/10) with a regimen of 1 g every 6 h; they showed an augmented Vd and CL_T_ and thus suggested the need for this high dose regimen in this subpopulation. In 2018 Li and Xie [17] reported some conflicting results assuming a 40% fT > MIC target. They performed Monte Carlo simulations based on 20 burn patients and showed a PTA of 100% for a regimen of 0.5 g every 6 h for MIC ≤ 2 mg/L, while higher doses were needed for higher MICs. These results were substantially confirmed in 2021 by Por et al. [42], who assumed the same target and performed Monte Carlo simulations based on patients who were undergoing CRRT (12 patients) and those who were not undergoing CRRT (11 patients). In their model, the target was more difficult to attain in patients experiencing ARC, or those who were placed on CRRT without a significant baseline renal impairment.

Finally, Machado et al. [15] performed a before/after study on the implementation of TDM as guidance for antibiotic treatment in burn patients. They included 42 patients treated with imipenem-cilastatin and showed a PTA of 100% for MIC ≤ 2 mg/L, but details on drug regimens were not provided. It is worth noting that CI is not recommended for imipenem-cilastatin due to stability issues [50].

#### Synthesis of the Findings concerning Drug Regimens for Imipenem-Cilastatin

Evidence for the use of imipenem-cilastatin in burn patients is limited. To achieve a target of 50–100% fT > MIC [21], a dose of 500 mg every 6 h should be sufficient, at least in patients with normal renal function and who are infected by pathogens with MIC ≤ 2 mg/L. Higher TDM-guided doses, namely 1 g every 6 h, may be needed for patients with ARC or higher MICs.

### 2.6. Aztreonam

The initial literature search found 48 studies, and 2 were selected after TIAB screening and full-text analysis. Evidence was consequently extremely limited. Friedrich et al. [13] studied the PK of aztreonam in 8 adult patients with severe burn injuries (mean burned TBSA of 49%) and showed that the PK of aztreonam was altered, particularly because of increased Vd, which was found to be 30% higher than that reported for other patient populations. Moreover, Vd was correlated with the extension and severity of burn injury. The other main PK parameter affected by burns was CL_T_; however, its alteration was independent from the extension and severity of the burn injury. The authors proposed an increase in the posology of aztreonam for burn patients, but suggested neither a specific PK/PD target nor a given drug regimen.

Falcone et al. [34] performed a population PK analysis coupled with Monte Carlo simulations of the association of ceftazidime-avibactam and aztreonam. In their sample of 41 critically ill patients (including 8 burn patients), they showed a high Vd and lower CL compared with previously available data on aztreonam. They confirmed that CL_R_ was the main PK parameter influencing the PK of aztreonam. Overall, they did not suggest any dosing adjustment for aztreonam to achieve a target of 100% fT > MIC. In the subpopulation of difficult-to-treat patients having high CL_CR_ and high MIC (CL_CR_ > 90 mL/m and MIC > 8 mg/L), a dosing regimen of 8 g in 24 h by CI (after a 2 g loading dose) was suggested. However, these recommendations concerned the entire patient population and could not be extrapolated specifically for burn patients.

#### Synthesis of the Findings concerning Drug Regimens for Aztreonam

Evidence for the use of aztreonam in burn patients is very limited, both in terms of number of studies and number of included patients. To achieve a target of 50–100% fT > MIC [34], a dose of 2 g of aztreonam every 8 h should be sufficient. After a loading dose, a dosing regimen of 8 g/24 h via CI may be more appropriate when treating severe infections, patients with high CL_CR_ or when there are strains with higher MICs.

### 2.7. New Beta-Lactam-Beta-Lactamase Inhibitor Combinations, Ceftaroline, Ceftobiprole and Cefiderocol

The initial literature search found 3 papers for new beta-lactam-beta-lactamase inhibitor combinations (BL/BLI), i.e., ceftolozane-tazobactam, ceftazidime-avibactam, meropenem-vaborbactam and imipenem-cilastatin-relebactam. One paper was selected after TIAB screening and full-text analysis: the previously mentioned paper by Falcone et al. [34] that focused on ceftazidime-avibactam (see sections on aztreonam). Given their PK analysis, which was coupled with Monte Carlo simulations focusing on critically ill patients, they suggested that standard or even lower doses of ceftazidme-avibactam could be sufficient, except in patients with a CL_CR_ between 6 and 15 mL/m. However, as was already emphasized, their sample included only 8/41 burn patients.

For all the other BL/BLI, as well as for ceftaroline, ceftobiprole and cefiderocol, we did not find any specific information on PK in burn patients.

#### Synthesis of the Findings concerning Drug Regimens for New Beta-Lactam-Beta-Lactamase Inhibitor Combinations, Ceftaroline, Ceftobiprole and Cefiderocol

The evidence for the use of these antibiotics in burn patients is insufficient to give any recommendations.

## 3. Discussion

We performed a systematic review of evidence on the pharmacology of broad-spectrum beta-lactams in the special population of burn patients. We decided to focus on these drugs for two main reasons. First, they are the cornerstone of antibiotic treatment in this setting, where MDR gram-negative pathogens are frequent, particularly *Pseudomonas aeruginosa*, but also MDR *Enterobacterales* and *Acinetobacter baumannii* [9,10]. Second, it is known that the PK/PD properties of these agents can significantly change in the population of burn patients [1,2,3,51], making the usual drug regimens potentially inappropriate.

Overall, we found that available studies were quite limited: they were heterogeneous in terms of methodologies and populations, and often included small sample sizes. The evidence regarding meropenem is more solid than that of other agents, with some well-conducted studies showing substantially concordant results [16,33,39,46]. Evidence is poorer (and somewhat discordant across studies) for piperacillin-tazobactam, ceftazidime, cefepime and imipenem-cilastatin, and very limited for aztreonam. Finally, the data are completely lacking for new beta-lactam-beta-lactamase inhibitor combinations ceftaroline, ceftobiprole and cefiderocol.

If we consider the classic PK parameters, such as Vd, CL and T1/2, there is a relative abundance of data, even if studies are not always concordant in their conclusions. Steele et al. [5] and Cota et al. [2] published two eminent reviews a few years ago on what is known about these classic PK parameters in burn patients. We believe that these two papers can be taken as references on this specific topic. Volume of distribution is often increased in burn patients, probably due to capillary leakage and fluid extravasation in the interstitial space [1,2,5]. The studies also reported a close relationship between CL_R_, CL_CR_ and the clearance of beta-lactams [13,26,31,32,47]. This is not surprising, considering that the majority of beta-lactams are renally cleared [52,53]. These two aspects (increased Vd and the close relationship with CL_CR_) are very relevant, since they can cause drug underexposure.

However, translating this evidence so that it informs clinical practice is very challenging. Scientific evidence is far more difficult to generalize when we take into account data on appropriate antibiotic regimens and their probability of achieving appropriate PK/PD targets. As already discussed, the debate about which PK/PD target may be the best for each class of antibiotics is still open and evolving [20,21]. This is particularly true for special populations, such as burn patients, where evidence is limited. This is reflected in the studies included in this review, in which PK/PD targets vary significantly, making generalizability of findings quite arduous. If we consider the included studies on piperacillin-tazobactam, meropenem and imipenem-cilastatin, the PK/PD targets vary widely from 40–50% fT > MIC up to 100% fT > 4 × MIC. Obviously, this has a huge impact on the conclusions that can be drawn about which drug regimens may be the best. Moreover, data about antibiotic penetration and tissue levels in injured and repairing tissues are extremely limited [49,54].

Given these important limitations, we tried to summarize the retrieved evidence about which regimens could be the best for different broad-spectrum beta-lactam antibiotics in burn patients. Overall, we suggest using high antibiotic doses, preferably via CI, after a loading dose (Table 2). Beta-lactams have a wide therapeutic window, with high concentration thresholds for toxicity [21], and this approach has already been suggested for the overall population of critically ill patients [52,53,55,56,57]. Continuous infusion is particularly needed for empiric treatments, since *P. aeruginosa* is frequent in this patient population [9,10], and it exhibits higher MICs compared with *Enterobacterales*, even in the absence of acquired mechanisms of resistance [58].

High doses and CI are necessary particularly in patients experiencing ARC. The role of ARC has been increasingly emphasized in recent literature as a major determinant of drug underexposure in critically ill patients [58]. ARC occurs frequently in burn patients. Overall, the median CL_CR_ across the studies included in this review was 129 mL/m, with 13 studies reporting a population with a mean CL_CR_ > 120 mL/m (Table 1). Despite this evidence, we found little data specifically addressing the topic of drug regimens in burn patents with ARC [33,37,43,45]. Considering these data and the existing literature on other subpopulations of critically ill patients [52,53,57], we can extrapolate the need for higher doses and CI in this patient population, particularly for piperacillin-tazobactam and the carbapenems. However, giving any specific indication about drug regimens for different degrees of ARC is currently unfeasible.

Another challenging scenario concerns patients with renal impairment, including patients treated with CRRT. It is already known from the overall population of critically ill patients that achieving adequate drug exposure can be challenging in these special subgroups [59,60,61,62]. In our systematic review, we found very little data about these subpopulations; the existing data mainly concerned imipenem-cilastatin [12,17,35,42,45,48]. Thus, it is not possible to extrapolate specific indications about drug regimens in burn patients experiencing renal failure or undergoing CRRT. However, we believe that in the case of CRRT the real need for dose reduction should be carefully assessed, since a full dose can be required based on factors such as CRRT characteristics (flow, type of membrane), antibiotic-protein binding, residual renal function, severity and site of infection, and MICs of the pathogens [61]. Considering these factors, dose reduction is probably unnecessary in the majority of burn patients treated with CRRT. Continuous infusion and TDM guidance are essential in this patient population.

Regarding obesity, data are lacking in the burn patient population. Some studies included a sample having high mean body weight or BMI [30,42,45,46], but it is impossible to draw any conclusions about specific drug regimens. Thus, we suggest applying what is already known about beta-lactam use in obese critically ill patients, particularly concerning the need for CI and TDM guidance [63].

The extension of burned TBSA is a major factor influencing drug exposure, since it is correlated with CL_T_, Vd and alteration in protein homeostasis [2]. Overall, the mean % of burned TBSA in the included studies was quite high (34%), but TBSA emerged as a determinant of drug exposure only in few studies concerning piperacillin-tazobactam and aztreonam [13,14], whereas data for meropenem were conflicting [46]. Although burn extension is surely a major factor to consider when choosing high dosing regimens of beta-lactam antibiotics, it is not possible to give any specific indications in this regard.

Therapeutic drug monitoring is having an ever-growing role in guiding beta-lactam use in critically ill patients, and its implementation in this patient population is now recommended [20]. Four studies explored the use of TDM guidance in burn patients [14,15,22,41] and showed that the PTA was higher in subgroups of patients whose treatment was guided by TDM. Although none of these studies found any significant impact of TDM on mortality or development of antibiotic resistance, they were not sufficiently powered to explore these outcomes. We believe that TDM should be implemented in the management of antibiotic treatment of burn patients, particularly in those patients with severe infections caused by pathogens with high MIC values, renal failure, CRRT, ARC or morbid obesity. TDM guidance is also essential in achieving more aggressive PK/PD targets, which are associated with the suppression of the emergence of antibiotic resistance [20].

We recognize that this systematic review has some limitations. We screened only MEDLINE; thus, some studies not indexed by that database may have been missed. TIAB screening and data extraction were performed by only one author. Moreover, dosing suggestions were based only on PK/PD targets suggested in the position paper by Abdul-Aziz et al. [21]. Different conclusions may have been reached if more ambitious targets had been considered, such as those recently suggested by Hong et al. [20].

## 4. Materials and Methods

We performed a systematic review of original articles that evaluated the pharmacological properties of broad-spectrum beta-lactam antibiotics in adult burn patients. The following antibiotics were considered: piperacillin-tazobactam, ceftazidime, cefepime, meropenem, imipenem-cilastatin, aztreonam, ceftolozane-tazobactam, ceftazidime-avibactam, meropenem-vaborbactam, imipenem-cilastatin-relebactam, ceftaroline, ceftobiprole and cefiderocol.

The systematic review was conducted in accordance with PRISMA guidelines [64].

The PICO questions were applied as follows: (a) Population: adult burn patients; (b) Intervention: evaluation of PK properties of one of the aforementioned antibiotics; (c) comparator: not relevant; (d) outcomes: evaluation of PK/PD indexes and attainment of pre-specified PK/PD targets.

The selection criteria (all the following criteria were needed) included: (a) studies reporting clinical data on the pharmacological properties of the aforementioned antibiotics; (b) studies in humans; (c) participants > 18 years old; (d) no restriction on burn severity or ward setting; (e) clinical studies (no in-vitro studies); (f) studies reporting original data, excluding pre-prints and conference abstracts; (g) all study designs, except narrative reviews; (h) studies written in English, French, Spanish or Italian.

The search was performed on MEDLINE, and it included research studies published between its inception and the 15th of January 2023. The search strings are detailed in Appendix A. First, we selected potentially relevant papers via a TIAB screening. Selected articles were further assessed for eligibility with a full-text analysis. Deduplication of selected references was performed using Zotero software (Version 6.0.18). Data extraction was realized in a pre-specified form and included: year of publication; first author; journal of publication; full reference; country where the study was conducted; language; study characteristics (including: study design, studied antibiotic(s), sample size); patient characteristics (including % of burned TBSA, weight, BMI, creatinine clearance—CL_CR_); target PK/PD parameter(s); and % of target attainment with the studied regimen(s). Excel (Microsoft Office Professional Plus 2013, Microsoft Corporation, Redmond, WA, USA) was used for data collection and a descriptive analysis was performed.

Based on the evidence found, we proposed (when feasible) a final, synthetic recommendation for each antibiotic about the regimen thought to have the best chance to attain therapeutically relevant PK/PD targets. Since no specific set of PK/PD targets were proposed for the population of burn patents, we used the PK/PD targets reported in the aforementioned position paper by Abdul-Aziz et al. as references [21] (Table 3).

## 5. Conclusions

In conclusion, we performed a systematic review of the pharmacology of beta-lactams in burn patients that focused on optimal drug regimes. Our findings emphasized the need for high antibiotic doses administered by CI in this population, and that implementing TDM-guided strategies could be worthwhile in this setting.

## 6. Future Directions

Many aspects of the burn patient population remain to be elucidated: validating established PK/PD targets; identifying antibiotics with the highest probability of PK/PD target attainment; defining which could be the best antibiotic regimens in special populations, such as patients with renal failure, patients undergoing CRRT, patients with ARC or obese patients; defining which could be the PK/PD target of novel beta-lactam/beta-lactamase inhibitor combinations and cefiderocol; and finally, how to implement TDM of these antibiotics in real-life scenarios and how best to assess its impact on clinical outcomes.

## Figures and Tables

**Table 1:** Studies included in the systematic review.

First Author	Target Antibiotic(s)	Country of the First Author	Year of Publication	Study Design	Number of Patients	Control Group	Mean % of Burned TBSA	Mean CL_CR_ (mL/m)	Mean Body Weight (Kg)	Suggested PK/PD Target(s)
Alshaer [22]	FEP, TZP, MEM	USA	2022	Before/after study	23 (post-intervention)	19 (pre-intervention)	25	119	Na	Cmin > MIC; Cmin > 4 × MIC
Aoki [23]	FEP	Japan	2010	Case report	1	Nap	46	163	Na	60–70% fT > MIC
Bonapace [11]	FEP	USA	1999	Clinical PK study + population PK model	12	Nap	36	135	84	60% T > MIC
Boucher [24]	IPM	USA	1990	Clinical PK study	11	Nap	43	105	71	None
Boucher [12]	IPM	USA	2016	Clinical PK study	10	Nap	23	In CRRT	Na	None
Bourget [25]	TZP	France	1995	Clinical PK study	10	Nap	41	120	78	100% T > MIC
Conil [26]	CAZ, FEP	France	2007	Clinical PK study	30	Nap	Na	Na	Na	Cmin > 4 × MIC
Conil [27]	CAZ	France	2007	Clinical PK study + population PK model	50	Nap	23	105	71	None
Conil [28] 05	CAZ	France	2007	Randomized controlled trial with clinical PK study	15	15	Na	Na	Na	Cmin > 4 × MIC
Conil [29]	CAZ	France	2013	Clinical PK study + population PK model	70	Nap	32	118	74	Steady state concentration of 40–100 mg/L
Corcione [16]	MEM	Italy	2020	Clinical PK study + population PK model	17	34	134	Na	BMI: 25 kg/m^2^	75% fT > MIC
Cotta [30]	MEM	Australia	2015	Case report	1	Nap	50	129	100	54% fT > MIC; 10% fT > 4–6 MIC
Dailly [31]	IPM	France	2003	Clinical PK study + population PK model	47	Nap	28	Na	75	None
Dailly [32]	CAZ	France	2003	Clinical PK study + population PK model	41	Nap	34	Na (mean serum creatinine: 75 mmol/l)	74	None
Doh [33]	MEM	South Korea	2010	Clinical PK study + population PK model	59	0	49	138	66	40% fT > MIC
Falcone [34]	ATM; CZA	Italy	2021	Clinical PK study + population PK model	8 burn patients, 41 total patients	Nap	Na	Na	BMI: 24 kg/m^2^	100% fT > MIC
Fournier [14]	TZP; MEM	Switzerland	2018	Monocentric, unblinded, randomized, controlled trial	19	19	20	Na	Na	100% fT > MIC
Friedrich [13]	ATM	USA	1991	Clinical PK study	8	Nap	49	Na	83	None
Gomez [35]	IPM	Brazil	2015	Clinical PK study + population PK model	51	36	Not specified, 36 patients with normal renal function and 15 with renal failure	68	Na	40% fT > MIC
Hallam [36]	MEM	UK	2010	Case report	1	Nap	52	Na	Na	100% fT > MIC
Jeon [37]	TZP	South Korea	2014	Clinical PK study + population PK model	5	Nap	35	132	67	50% fT > MIC
Le Floch [38]	CAZ, IPM	France	2009	Clinical PK study	Number of patients not specified; 120 samples	Nap	32	153	76	100% fT > 4xMIC
Li [17]	IPM	Belgium	2018	Clinical PK study + population PK model	20	Nap	Na	Na	Na	40% fT > MIC
Machado [15]	TZP, IPM, MEM	Brazil	2017	Before/after study	77	63	31	Na	81	100% fT > MIC for TZP; 60% fT > MIC for IPM and MEM
Messiano [39]	MEM	Brazil	2022	Clinical PK study	15	Nap	33	100	BMI: 24 kg/m^2^	100% fT > MIC
Olbrisch [40]	TZP	Germany	2018	Prospective cohort study with PK study + population PK model	20	16	31	Na	80	100% fT > 4xMIC
Patel [41]	TZP; MEM	Australia	2012	Clinical PK study	50 (6 on TZP, 1 on MEM)	Nap	17	86	Na	100% fT > MIC; 100% fT > 4 × MIC
Por [42]	IPM	USA	2021	Clinical PK study + population PK model	12 in CRRT	11 not in CRRT	43	132	98	40% fT > MIC
Ramon-Lopez [43]	MEM	UK	2015	Clinical PK study + population PK model	12	Nap	41	137	83	40% T > MIC; 60% T > MIC; 80% T > MIC
Sampol [44]	FEP	France	2000	Clinical PK study	6	Nap	32	123	Na	None
Selig [45]	TZP	USA	2022	Case-control study + population PK model	5	14	38	210	103	50% fT > MIC; 100% fT > MIC; 100% ft > 4 × MIC
Selig [46]	MEM	USA	2022	Case-control study + population PK model	11	12	33	150	88	40% fT > MIC; 99% fT > MIC
Shikuma [47]	TZP	USA	1990	Clinical PK study	9	Nap	Na	90–120	Na	Not specified
Torian [48]	TZP	USA	2023	Case report	1	Nap	3.2	>120	Na	100% T > MIC
Walstad [49]	CAZ	Norway	1998	Clinical PK study with measurement of tissue concentration	8	Nap	20–80	Na	Na	None

ATM: aztreonam. CAZ: ceftazidime. CL_CR_: creatinine clearance. Cmin: trough concentration. CRRT: continuous renal replacement therapy. CZA: ceftazidime-avibactam. FEP: cefepime. fT > MIC: duration of time (T) that the free drug concentration remained above the MIC during a dosing interval. IPM: impenem-cilastatin. MEM: meropenem. MIC: minimum inhibitory concentration. Na: not available. Nap: not applicable. PK/PD: pharmacokinetic/pharmacodynamic. TBSA: total body surface area. TZP: piperacillin-tazobactam.

**Table 2:** Synthesis of findings concerning drug regimens.

Antibiotic	Advisable Drug Regimens in Burn Patients without Renal Impairment	Suggested Modalities of Preparation and Administration [50]
Ceftazidime	At least 1 g every 4 h or CI of 6 g/24 h (with loading dose)	Using syringe pump: 2 g in 50 mL of normal saline (0.9%) or glucose solution (5%), over 8 h, 3 times a day
Cefepime	2 g every 8 h; consider CI of 6 g/24 h (with loading dose), particularly for severe infections or high MICs	Using syringe pump: 2 g in 50 mL of normal saline (0.9%) or glucose solution (5%), over 8 h, 3 times a day
Piperacillin-tazobactam	18 g/24 h CI (with loading dose). Higher doses may be needed for patients with ARC or high MICs	Using syringe pump: 4.5 g in 50 mL of normal saline (0.9%) or glucose solution (5%), over 6 h, 4 times a day
Meropenem	6 g/24 h CI (with loading dose). Higher doses may be needed for patients with ARC	Using syringe pump: 2 g in 50 mL of normal saline (0.9%), over 8 h, 3 times a day
Imipenem-cilastatin	500 mg every 6 h; 1 g every 6 h if ARC or MIC > 2 mg/dL	500 mg in 100 mL of normal saline (0.9%), in 30 min, 4 times a day; 1 g in 250 mL of normal saline (0.9%), over 1 h, 4 times a day. Continuous infusion not recommended due to stability issues
Aztreonam	2 g every 8 h or 6–8 g in CI (particularly for high CL_CR_ or high MICs)	2 g in 100 mL of normal saline (0.9%), in 30 min, 3 times a day; Using syringe pump: 3 g in 50 mL of normal saline (0.9%), over 12 h, 2 times a day
Ceftaroline, ceftobiprole, ceftolozane-tazobactam, meropenem-vaborbactam, imipenem-cilastatin-relebactam, cefiderocol	No specific data on burn patients. Standard regimens: - ceftaroline: 0.6 g every 8 h - ceftobiprile: 0.5 g every 8 h - ceftolozane-tazobactam: 3 g every 8 h - meropenem-vaborbactam: 4 g every 8 h - imipenem-cilastatin-relebactam: 1.25 g every 6 h - cefiderocol: 2 g every 6 h	- ceftaroline *: 0.6 g in 50–250 mL of normal saline (0.9%) over 2 h, 3 times a day - ceftobiprole *: 0.5 g in 250 mL of normal saline (0.9%) or glucose solution (5%), over 2 h, 3 times a day - ceftolozane-tazobactam *: 3 g in 100 mL of normal saline (0.9%) or glucose solution (5%), over 3 h, 3 times a day - meropenem-vaborbactam *: 4 g in 250 mL of normal saline (0.9%), over 3 h, 3 times a day - imipenem-cilastatin-relebactam: 1.25 g in 100 mL of normal saline (0.9%), over 30 min, 4 times a day - cefiderocol *: 2 g in 100 mL of normal saline (0.9%), over 3 h, 3 times a day * Continuous infusion may be proposed
Ceftazidime-avibactam	Insufficient data. Standard regimen: 2.5 g every 8 h	2.5 g in 50 mL of normal saline (0.9%), over 2 h, 3 times a day. Continuous infusion may be proposed.

ARC: augmented renal clearance. CI: continuous infusion. CL_CR_: creatinine clearance. MIC: minimum inhibitory concentration.

**Table 3:** Standard drug dosages, EUCAST Clinical Breakpoints (v. 13.1) and suggested PK/PD targets.

Antibiotic	Dosages Used by EUCAST to Define Breakpoints [65]		EUCAST Clinical Breakpoint Tables v. 13.1 (mg/L)		Suggested PK/PD Target [21]	
	Standard Dosage	High Dosage	S≤	R>	Pre-Clinical PK/PD Target for Efficacy	Clinical PK/PD Target for Efficacy
Ceftazidime	1 g × 3	2 g × 3 or 1 g × 6	*Ent*: 1 *Pa*: 0.001	*Ent*: 4 *Pa*: 8	60–70% fT > MIC	40–100% fT > MIC
Cefepime	1 g × 3 or 2 g × 2	2 g × 3. For severe *P. aeruginosa* infections: 2 g in 4 h × 3	*Ent*: 1 *Pa*: 0.001	*Ent*: 4 *Pa*: 8	60–70% fT > MIC	40–100% fT > MIC
Piperacillin-tazobactam	4.5 g × 4 or 4.5 g in 4 h × 3	4.5 g in 3 h × 4	*Ent*: 8 *Pa*: 0.001	*Ent*: 8 *Pa*: 16	50% fT > MIC	50–100% fT > MIC
Meropenem	1 g × 3	2 g in 3 h × 3	*Ent*: 2 *Pa*: 2	*Ent*: 8 *Pa*: 8	40% fT > MIC	50–100% fT > MIC
Imipenem-clinastatin	0.5 g × 4	1 g × 4	*Ent*: 2 *Pa*: 0.001	*Ent*: 4 *Pa*: 4	40% fT > MIC	50–100% fT > MIC
Aztreonam	1 g × 3	2 g × 4	*Ent*: 1 *Pa*: 0.002	*Ent*: 4 *Pa*: 16	/	50–100% fT > MIC [34]
Ceftolozane-tazobactam	1.5 g × 3	3 g × 3	*Ent*: 2 *Pa*: 4	*Ent*: 2 *Pa*: 4	/	/
Ceftazidime-avibactam	2.5 g in 2 h × 3		*Ent*: 8 *Pa*: 8	*Ent*: 8 *Pa*: 8	/	/
Meropenem-vaborbactam	4 g in 3 h × 3		*Ent*: 8 *Pa*: 8	*Ent*: 8 *Pa*: 8	/	/
Imipenem-cilastatin-relebactam	0.75 g × 4	none	*Ent*: 2 *Pa*: 2	*Ent*: 2 *Pa*: 2	/	/
Ceftaroline	0.6 g × 2	0.6 g × 3	*Ent*: 0.5	*Ent*: 0.5	/	/
Ceftobiprole	0.5 in 2 h × 3	none	*Ent*: 0.25	*Ent*: 0.25	/	/
Cefiderocol	2 g in 3 h × 3	none	*Ent*: 2 *Pa*: 2	*Ent*: 2 *Pa*: 2	/	/

*Ent: Enterobacterales*. fT > MIC: duration of time (T) that the free drug concentration remains above the MIC during a dosing interval. MIC: minimum inhibitory concentration. *Pa: Pseudomonas aeruginosa*. PK/PD: pharmacokinetic/pharmacodynamics. R: resistant. S: susceptible.

## Data Availability

Additional data available on request from corresponding author.

## Appendix A. Search Strings

**Table A1:** Search strings. Search date: 15 January 2023.

Search Strings	Number of Hits	Paper after TIAB and Full-Text Analysis *
(“burn” [tiab] or “burns” [tiab] or “burned” [tiab]) and “piperacillin” [tiab]	127	10
(“burn” [tiab] or “burns” [tiab] or “burned” [tiab]) and “ceftazidime” [tiab]	168	7
(“burn” [tiab] or “burns” [tiab] or “burned” [tiab]) and “cefepime" [tiab]	51	5
(“burn” [tiab] or “burns” [tiab] or “burned” [tiab]) and “meropenem” [tiab]	131	11
(“burn” [tiab] or “burns” [tiab] or “burned” [tiab]) and “imipenem” [tiab]	270	8
(“burn” [tiab] or “burns” [tiab] or “burned” [tiab]) and “aztreonam” [tiab]	48	2
(“burn” [tiab] or “burns” [tiab] or “burned” [tiab]) and “avibactam” [tiab]	5	1
(“burn” [tiab] or “burns” [tiab] or “burned” [tiab]) and “vaborbactam” [tiab]	2	0
(“burn” [tiab] or “burns” [tiab] or “burned” [tiab]) and “ceftolozane” [tiab]	2	0
(“burn” [tiab] or “burns” [tiab] or “burned” [tiab]) and “relebactam” [tiab]	1	0
(“burn” [tiab] or “burns” [tiab] or “burned” [tiab]) and “ceftaroline” [tiab]	3	0
(“burn” [tiab] or “burns” [tiab] or “burned” [tiab]) and “ceftobiprole” [tiab]	2	0
(“burn” [tiab] or “burns” [tiab] or “burned” [tiab]) and “cefiderocol” [tiab]	3	0
Total *	813	44

TIAB: Title and abstract screening. * This number is different compared to that reported in Figure 1 and Table 2 (where we reported 35 selected studies) because some papers targeted more than one antibiotic. In this table, these studies are listed more than once, i.e., for each particular antibiotic.
